# Patient-centered care and patient satisfaction: Validating the patient-professional interaction questionnaire in China

**DOI:** 10.3389/fpubh.2022.990620

**Published:** 2022-11-07

**Authors:** Tao Han, Sisi Li, Xueyuan Li, Chenhao Yu, Jiahui Li, Tiantian Jing, Mayangzong Bai, Yue Fang, Kun Qian, Xiaoyan Li, Huigang Liang, Zhiruo Zhang

**Affiliations:** ^1^School of Public Health, Shanghai Jiao Tong University School of Medicine, Shanghai, China; ^2^Kunshan Huaqiao People's Hospital, Kunshan, China; ^3^Department of Business Information and Technology, Fogelman College of Business and Economics, University of Memphis, Memphis, TN, United States

**Keywords:** patient-centered care, PCC, patient-professional interaction questionnaire, PPIQ, patient satisfaction, Chinese patient satisfaction, China

## Abstract

**Objective:**

To introduce patient-centered approach in China and to relate it with Chinese patient satisfaction *via* validating the Chinese version of Patient-Professional Interaction Questionnaire (PPIQ-C).

**Design:**

This cross-sectional survey was conducted through face-to-face interviews from June to September in 2019. Participants rated their patient-centered care experience *via* the 16-item translated PPIQ, their experience of the received medical service, and their overall satisfaction.

**Setting:**

Kunshan Huaqiao People's Hospital in Jiangsu, China.

**Participants:**

A total of 230 participants (87 males and 143 females; 108 outpatients and 122 inpatients).

**Results:**

PPIQ-C exhibited acceptable psychometric properties. Data revealed a single factor model of the 16 PPIQ-C items [$\chi_{\left(4\right)}^{2}$ = 12.394, *p* = 0.823, CFI = 1.000, TLI = 1.019, RMSEA = 0.000, SRMR = 0.032] had a superior model fit over the original first-order with four correlated factors and the second-order structures. The overall reliability was excellent (McDonald's ω = 0.975). In terms of patient satisfaction, process, treatment quality, and communication significantly predicted patient satisfaction, while environment, staff attitude, and medical ethics did not [*R*^2^ = 0.427, *F*_(6)_ = 24.887, *p* < 0.001]. Most importantly, the total score of PPIQ-C predicted patient satisfaction above and beyond the above-mentioned medical service perspectives (*B* = 0.595, *SE* = 0.207, *p* = 0.004). Finally, the constructive effect of PCC on patient satisfaction was stronger for departments of Pediatrics than Surgery.

**Conclusions:**

The Chinese version of the PPIQ scale (PPIQ-C) exhibited acceptable psychometric properties. Yet the distinction among the four factors was not supported, suggesting potential difference(s) across cultures. Patient-centered care (PCC), reflected by the overall PPIQ-C score, predicted overall patient satisfaction above and beyond other medical service perspectives. Adopting PCC approach in appropriate situations will probably advance the development of performance evaluation systems in China, thus improving the overall health care and patient satisfaction.

## Background

Over the last two decades there has been an increasing focus on supporting people to be more involved in their care and in tailoring services around the needs of individuals (1). Engaging people in their health and care is now recognized as a key factor of developing healthcare of the highest quality (2). The concept of patient-centered care (PCC) was first coined in 1986 by the Picker/Commonwealth Patient-Centered Care Program (3). It is an approach that sees patients as equal partners in planning, developing, and assessing care to make sure it is most appropriate for their needs (4). It emphasizes more on patient participation and involvement, the relationship between the patient and the healthcare professional, and the environment where care is delivered (5). Over past decades, the concept has evolved and been applied to various aspects of healthcare. Traditional healthcare approach aims at providing healthcare to the majority of the population; hence people are expected to adapt to the routines and practices that the service providers deem most appropriate. In contrast, person-centered care requires that services be more flexible and meet people's needs in the way that best serves them (6). Previous studies widely supported that patient-centered care can help improve outcomes and reduce the burden on health services. It constitutes one of the six pillars in medical service quality assessment (7), and has been widely used in developed countries, especially in the field of patient satisfaction evaluation (8).

Patient satisfaction is a critical indicator in the evaluation of healthcare service, which is commonly used in performance appraisal of medical institutions (9, 10). The evaluation of patient satisfaction allows healthcare providers to identify service factors that needs improvement (11). It also enables policymakers to understand patients' needs and to make strategic plans for high-quality services (12). However, since patient satisfaction is a complex and multidimensional concept, its potential determinants evaluation methods differ greatly across studies (13, 14). Current studies on patient satisfaction mainly focus on the medical quality, environment, service attitude, communication, and other indicators to carry out investigations (15). Moreover, it is widely supported that PCC contributes to patient satisfactions in various medical settings (16). PCC benefits not only the concordance between care provider and patient on treatment plans, but also healthcare outcomes (17, 18).

However, PCC receives relatively less attention in developing countries, for example, China (19, 20). Due to its huge population base, the per capita medical treatment time is seriously insufficient in China, thus medical activities have long been dominated by professionals. It was somewhat reasonable that patient orientated dimensions such as compassion and patient involvement did not receive sufficient attention in Chinese medical settings considering its unevenly distributed medical resources. Said which, major innovations and breakthroughs brought by Chinese large-scale healthcare reforms have been redirecting its healthcare policies and practices toward patient-centering (10, 21).

In light of the insufficient attention to PCC in patient satisfaction evaluation in China (20, 22), it is beneficial to introduce the concept of PCC and its corresponding assessment tool. The Patient-Professional Interaction Questionnaire (PPIQ) is a well-established patient-perspective assessment of PCC (23) with four key factors including, (1) effective communication (EC), which plays a fundamental role in asking questions from and listening attentively to patients in order to deliver healthcare in clear, respectful, and efficient ways; (2) interest in patient's agenda (IPA), which highlights the importance that not only symptoms and courses but also patients' feelings, desires, and expectations should be taken into medical consideration, (3) empathy (E), which encourages both emotionally and cognitively taking patients' perspectives to engage patients as alliance in healthcare delivery, and (4) patient involvement in care (PIC), which engages patients in informed and collaborative decision-making for treatment options (24). Studies revealed that PPIQ outperformed other PCC relevant questionnaires such that it was timesaving, easy to administer, free of social desirability, and most importantly, theory-grounded, valid, and reliable (23). PPIQ has been applied to both traditional and novel medical settings (e.g., remote monitoring of healthcare delivery in COVID-19) and in both western and non-western countries (25, 26).

To briefly summarize, this study provides a clear and comprehensive survey of the influence of PCC on patient satisfaction in China *via* introducing the translated, Chinese version of PPIQ (namely, the PPIQ-C). A conventional procedure was conducted to translate the PPIQ into Chinese, and the psychometric properties of the PPIQ-C were examined according to its theoretical structure. It was expected that PPIQ-C could serve as suitable assessment of PCC in Chinese medical settings, and it could predict Chinese patients' satisfaction of their medical experiences among other factors.

## Methods

### Study design and sample

A cross-sectional survey was conducted through face-to-face interviews from June to September in 2019. A total of 230 participants (87 males and 143 females; 108 outpatients and 122 inpatients) were recruited *via* convenient sampling from the a local second-grade, comprehensive hospital in the Jiangsu, China. The inclusion criteria were outpatients or inpatients of the hospital. Patients who were unable to make an objective and rational assessment because of illness or psychological disorder were excluded. Five respondents were excluded from the analyses regarding patient satisfaction because of incomplete answers in medical perspectives. All eligible participants who consented to participate were informed about the study's purpose and procedures and were asked to respond to the e-survey on tablets during the interview.

### Materials

#### Patient-professional interaction questionnaire Chinese version (PPIQ-C)

Participants rated their patient-centered care experience *via* the 16-item PPIQ-C (1 = *quite disagree* to 5 = *quite agree*). The original PPIQ (23) was translated to Chinese by a senior postgraduate student in Public Health, and then back-translated to English by another two senior postgraduate students. The translated versions were then compared to the original version to check for inconsistency, if any. And then, experts in Public Health and Social Psychology were consulted to evaluate the feasibility, accuracy, and readability. This procedure was repeated until the translated version was deemed satisfactory by all authors. PPIQ-C consists of four factors (four items each), namely effective communication (EC; e.g., “The doctor sent me a clear message”), interest in patient's agenda (PIA; e.g., “The doctor paid attention to my medical needs”), empathy (E; e.g., “The doctor could understand my emotions”), and patient involvement in care (PIC; e.g., “The doctor allowed me to express my opinion”). Scores were averaged such that higher scores represent higher provision of each factor, and hence, overall patient-centered care.

#### Patient experience and overall satisfaction

Participants rated their experience of the received medical service *via* 12 face-valid questions (two for each factor), including hospital environment, treatment process, treatment quality, staff attitude, doctor-patient communication, and medical ethics derived from national patient-perspective of care research (1 = *quite disagree* to 5 = *quite agree*) (21, 27). Each medical perspective was calculated by taking the average value of the corresponding two questions (see Supplementary materials for details). Finally, participants rated their overall satisfaction by the question “How was your overall satisfaction?” (1 = *not at all* to 10 = *extremely*).

Finally, participants reported the medical departments they visited (Pediatrics, Internal medicine, Surgery, or Obstetrics and Gynecology), and provided their sociodemographic information, including gender (0 = *female* and 1 = *male*), age (0 = *18 years or below*, 1 = *19–30 years*, 2 = *31–40 years*, 3 = *41–50 years*, 4 = *51–60 years*, and 5 = *61 years or above*), marital status (0 = *unmarried* and 1 = *married*), education (0 = *below high school*, 1 = *high school*, 2 = *collage degree*, 3 = *bachelor's degree*, and *4* = *master's degree or above*), as well as annual income (0 = ¥*50,000 or below*, 1= ¥*50,001–100,000*, 2 = ¥*100,001–200,000*, 3 = ¥*200,001–300,000*, or 4 = ¥*300,000 or above*).

### Analytical scheme

Reliability was tested by McDonald's ω in Jamovi 2.2.5.CFA was adopted to test the same structure as the original PPIQ (i.e., a first-order model with 4 correlated factors and a second-order model) in the R lavaan package with Diagonally Weighted Least Square (DWLS) estimator (28, 29). Multiple goodness-of-fit indices were adopted: χ^2^ and Satorra-Bentler scaled χ^2^ (S-B χ^2^); ratio of S-B χ^2^ to its degree of freedom (cutoff ≤5) (30); Root Mean Square Error of Approximation (RMSEA, cutoff <0.080) (31); Comparative Fit Index and Tucker-Lewis Index (CFI and TLI, cutoff ≥ 0.900); and Standardized Root Mean Square Residual (SRMR; cutoff < 0.080) (32–34). Average variance extracted (AVE) was adopted to evaluate discriminant validity across the four factors. Square-roots of AVE values larger than any correlation among any pair of latent constructs suggests good discriminant validity. Other statistical analyses were performed in SPSS 22.0. Multiple linear regressions were conducted to examine the associations among patient-centered care and patients' medical experiences and satisfaction amongst different medical departments.

## Results

A total of 230 participants were enrolled in this study. Data (identity information masked) and exemplar syntax can be found here: https://osf.io/n46ze/. Table 1 presents the sociodemographic characteristics of all the participants. Female participants made up 62.2 percent of the total sample. In terms of age, 93 of the participants are aged between 52 and 60, accounting for 40.4%. Among all participants, 83.0% were married and 39.1% had a master's degree or above. Further, 43.6% of the participants' annual incomes were below 50,000 Chinese yuan and 40.7% had annual incomes >200,000 Chinese yuan.

**Table 1:** Sociodemographic characteristics (*n* = 230).

		***n***	**Proportion (%)**
Sex	Male	87	37.8
	Female	143	62.2
Age	≤18	46	20.0
	19–30	19	8.3
	31–40	19	8.3
	41–50	36	15.7
	51–60	93	40.4
	≥61	17	7.4
Marriage	Unmarried	39	17.0
	Married	191	83.0
Education	<High school degree	15	6.5
	High school degree	36	15.7
	College degree	37	16.1
	Bachelor's degree	52	22.6
	≥Master's degree	90	39.1
Annual income	≤¥50,000	75	32.6
	¥50,001–100,000	10	4.3
	¥100,001–200,000	17	7.4
	¥200,001–300,000	70	30.4
	≥¥300,000	58	25.2

### Psychometric characteristics of the PPIQ-C

**Figure 1:**
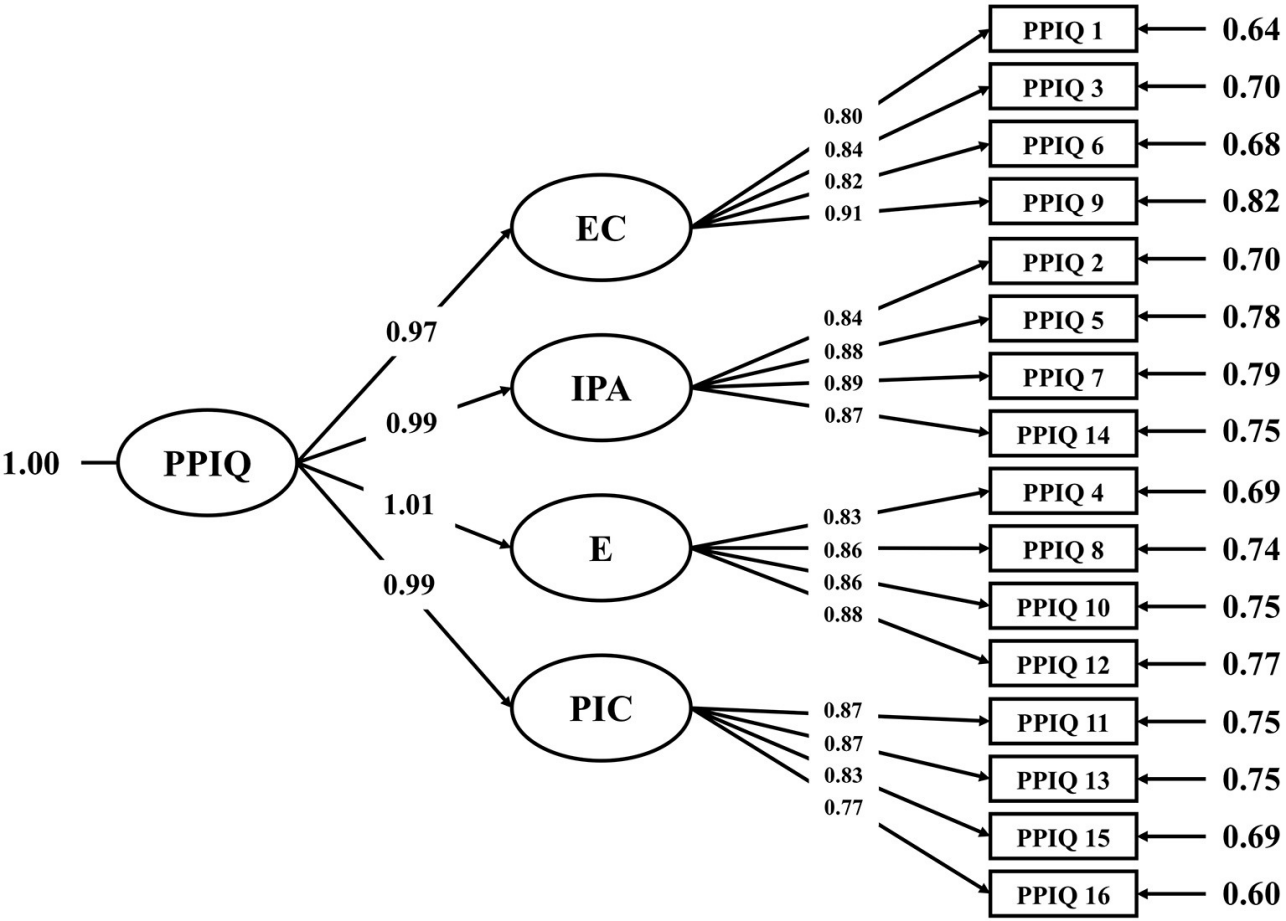
Measurement model of standardized PPIQ-C parameters. PPIQ-C, patient-medical staff interactive questionnaire; EC, Effective communication; IPA, Interest in patient's agenda; E, Empathy; PIC, Patient involvement in care.

Confirmatory factor analysis was adopted to examine the validity of the PPIQ-C scale. A first-order and a second-order four-factor [i.e., effective communication (EC), interest in patient schedule (IPA), empathy (E), and patient involvement in care (PIC)] models were compared in terms of the model fit. Fit indices were reported in Table 2, and parameter estimations were shown in Figure 1. Results suggested that both models fitted the data well and equivalently. To further examine the discriminant validity of the four factors, correlations of the factor scores were compared against the square roots of the AVE. Results suggested that the Fornell-Larcker Criterion was not met such that the square-roots of the AVE were smaller than the correlations among the factors. Considering this lack of discriminant validity, an additional CFA was conducted to examine a single factor model (see Model 3 in Table 2) and to compare its model fit against Model 2. Results suggested that Model 3 fitted the data well, and most importantly, it was not statistically different from Model 2. Scores for both the overall questionnaire and the four factors were computed. Internal consistency was assessed as well, both the overall questionnaire (McDonald's ω = 0.975) and the four factors exhibited satisfactory reliability (Table 3).

**Table 2:** Fit indices of the measurement models.

**Fit indices**	**Model 1**	**Model 2**	**Model 3**
*x^2^*	10.810	10.870	12.394
*Δ x^2^*	–	0.061	1.523
*DF*	98	100	104
*ΔDF*	–	2	4
S-B *x^2^*	0.110	0.109	0.119
ΔS-B *x^2^*	–	0.030	0.381
*p*	–	0.970	0.823
CFI	1.000	1.000	1.000
TLI	1.020	1.020	1.019
RMSEA	0.000	0.000	0.000
SRMR	0.030	0.030	0.032

Model 1 is the first-order model with four correlated factors; Model 2 is the second-order model; and Model 3 is the single factor model.

**Table 3:** Mean, SD, reliability, and inter-correlation of PPIQ-C factors (*n* = 230).

	***M***	***SD***	**McDonald's ω**	**1**	**2**	**3**	**4**
1. EC	4.29	0.57	0.910	(0.723)			
2. IPA	4.19	0.62	0.925	0.854	(0.753)		
3. E	4.18	0.64	0.918	0.871	0.899	(0.729)	
4. PIC	4.17	0.65	0.906	0.811	0.850	0.849	(0.686)
Overall score	4.21	0.59	0.975	–	–	–	–

The four factors correlated significantly with each other at p < 0.01. Values on the diagonal represent the square roots of AVE.

### Using PPIQ-C to predict patient satisfaction

PPIQ-C score was then applied to predict patient satisfaction. Three multiple linear regression models were examined. Model 1 included only medical service perspectives evaluated by patients as the predictors of patient satisfaction. Model 2 further included the PPIQ-C scores to examine the prediction of patient-centered care on patient satisfaction above and over the included medical service perspectives. Bi-variate correlation among all the six medical perspectives and the overall PPIQ-C scores has been shown in Table 4. Finally, model 3 examined whether the prediction of patient-centered care varied across different medical departments. All continuous predictors were centered.

**Table 4:** Bi-variate correlation among all the six medical perspectives and the overall PPIQ-C scores (*n* = 225).

	**1**	**2**	**3**	**4**	**5**	**6**	**7**
1. Environment	–						
2. Process	0.525	–					
3. Treatment quality	0.487	0.699	–				
4. Staff attitude	0.472	0.702	0.735	–			
5. Communication	0.541	0.727	0.809	0.767	–		
6. Medical ethics	0.527	0.687	0.639	0.602	0.695	–	
7. PCC	0.483	0.708	0.730	0.720	0.753	0.694	–

All correlations were significant at p < 0.01.

Results were summarized in Table 5. Process, treatment quality, and communication significantly predicted patient satisfaction, while the rest of medical perspectives did not (*R*^2^ = 0.427, *F* (6) = 24.887, *p* < 0.001). Specifically, patient satisfaction increased as the evaluation of process, treatment quality, and communication perspectives increased. When PPIQ-C score was further included in the model, it significantly predicted patient satisfaction above and beyond other medical service perspectives (*B* = 0.595, *SE* = 0.207, *p* = 0.004; *R*^2^ = 0.032, *F* (*df*) = 11.834, *p* < 0.001). Notably, treatment quality was no longer significant (*B* = 0.231, *SE* = 0.177, *p* = 0.192). Including PPIQ-C score further explained 3.3% of the total variance of patient satisfaction (*f*^2^ = 0.033). Finally, three dummy variables for departments [Internal medicine (IM), Surgery (S), Obstetrics and Gynecology (OG); 1 = *True*, and 0 = *False*] and their product terms with PPIQ-C scores were included (*F* = 13.949, *p* = 0.000; *f*^2^ = 0.026). Results suggested that the association of PPIQ-C score and patient satisfaction was only different between Surgery and Pediatrics departments (*B* = −0.768, *p* = 0.040); no statistical difference was found amongst other departments (*B*s < −0.347, *p*s > 0.299), such that 1 unit increase in PCC scores additionally brought 0.768 units increase in patient satisfaction in Pediatrics than in Surgery.

**Table 5:** Medical service perspectives and patient-centered care in patient satisfaction.

	**Model 1**				**Model 2**				**Model 3**			
	***B***	***SE***	***p***	**95% CI**	***B***	***SE***	***p***	**95% CI**	***B***	***SE***	***p***	**95% CI**
Environment	0.019	0.070	0.783	[−0.119, 0.158]	0.011	0.069	0.878	[−0.126, 0.147]	0.013	0.072	0.857	[−0.129, 0.115]
Process	**0.580**	**0.123**	**0.000**	**[0.337, 0.823]**	**0.534**	**0.122**	**0.000**	**[0.293, 0.775]**	**0.473**	**0.128**	**0.000**	**[0.220, 0.726]**
Treatment quality	**0.354**	**0.174**	**0.043**	**[0.011, 0.698]**	0.231	0.177	0.192	[−0.117, 0.580]	0.090	0.189	0.632	[−0.282, 0.463]
Staff attitude	−0.220	0.166	0.188	[−0.548, 0.108]	−0.303	0.166	0.070	[−0.630, 0.025]	−0.252	0.180	0.163	[−0.606, 0.103]
Communication	**0.664**	**0.194**	**0.001**	**[0.281, 1.047]**	**0.509**	**0.199**	**0.011**	**[0.117, 0.900]**	**0.462**	**0.207**	**0.027**	**[0.053, 0.871]**
Medical ethics	−0.075	0.113	0.506	[−0.298, 0.148]	−0.141	0.114	0.215	[−0.365, 0.083]	−0.064	0.120	0.595	[−0.301, 0.173]
PCC	–				**0.595**	**0.207**	**0.004**	**[0.187, 1.002]**	**1.081**	**0.324**	**0.001**	**[0.442, 1.719]**
IM	–				–				1.122	1.388	0.420	[−1.615, 3.860]
S	–				–				2.962	1.581	0.062	[−0.156, 6.080]
OG	–				–				0.670	1.416	0.637	[−2.123, 3.463]
IM × PCC	–				–				−0.346	0.333	0.300	[−1.003, 0.311]
S × PCC	–				–				**−0.768**	**0.372**	**0.040**	**[−1.501, −0.035]**
OG × PCC	–				–				−0.278	0.335	0.408	[−0.939, 0.383]
(Model summary)	***F***_**(6;200)**_ = 24.887, ***p*** < 0.001; ***R***^**2**^ = 0.427; ***f***^**2**^= 0.745				Δ***F***_**(7;199)**_ = 11.834, ***p*** < 0.001; Δ***R***^**2**^ = 0.032; Δ***f***^**2**^= 0.033				Δ***F***_**(13;193)**_ = 1.549, ***p*** < 0.001; Δ***R***^**2**^ = 0.025; Δ***f***^**2**^= 0.026			

n = 225. PCC, patient-centered care estimated by PPIQ-C. Medical departments were transformed into three dummy variables with Pediatrics (n = 46) being the reference group, where IM = Internal medicine (n = 53), S = Surgery (n = 48), OG = Obstetrics, and Gynecology (n = 65); dummy coding scheme: 1 = True, and 0 = False. Effects in bold were statistically significant.

## Discussion

This study translated and examined the psychometric properties of the Chinese version of the PPIQ scale (PPIQ-C), and then applied patient-centered care reflected by the PPIQ-C score to predict patient satisfaction. To summarize, the PPIQ-C exhibited excellent internal consistency as well as theorized dimensionality. In addition, patient-centered care, reflected by the PPIQ-C scores, predicted overall patient satisfaction above and beyond other medical service perspectives.

### Psychometric properties of the PPIQ-C

Although the first-order and the second-order model structures, as proposed by the original study (23), both fitted data well, the discriminant validity for the four factors were not statistically supported. Alternatively, a single factor model was adopted such that the factors were merged together (35), which exhibited satisfactory reliability and validity. The lack of discriminant validity could be the joint effect of the limited visiting time that Chinese doctors can allocate to each patient given China's large population, and the holistic but not analytical cognitive style of Asian cultures (36). In such circumstances, judgments of patient centered care delivery might rely heavily on overall impressions, and thus, resulting in highly correlated factors. Nevertheless, future studies should explore whether discriminant validity can be established for longer patient-doctor interaction (e.g., in inpatients).

Implication of factor scores was still discussed here as it could offer practical advice for local medical institutions to improve their patient centered care deliveries. As for individual scores, EC factor scored the highest across the current sample, which reflects the perceived extent to which professionals could allow patients to detail symptoms and maintain respect during visiting. This corroborates well with the extensive attention to communication skills in the standardized medical training programs in China. It also corroborates well with the growing emphasis of doctor-patient communication in job performance evaluation of professionals in China over the past decades. In contrast, PIC was rated as the lowest by patients. On the base of Chinese large population, it is not practical for medical professionals, especially those employed at large-scale, public hospitals, to involve patients to a large extent in medical decision-making. Therefore, it was reasonable that PIC received lower recognition compared to the other factors.

### PCC, patient experience, and overall satisfaction

Results suggested that treatment process, treatment quality and communication positively predicted patients' overall satisfaction. It is noteworthy that among the three factors communication exhibited the greatest prediction. This result aligns well with previous investigations in China (37). However, other medical service perspectives (i.e., hospital environment, staff attitude, and medical ethics) did not predict patient satisfaction. In contrast, another study found that hospital environment and facilities were more strongly related to overall satisfaction for rural respondents than for urban ones (38), which implies the diversity of medical satisfaction evaluation.

More importantly, when PCC was further included, it significantly and positively predicted overall satisfaction above and beyond other medical factors that were widely explored in patient satisfaction research (25, 26). It was especially noteworthy that PCC and doctor-patient communication independently contributed to overall satisfaction. Patient-centered care is characterized by three key factors of compassion, dignity, and respect, which are implemented *via* shared decision making, supporting self-management, and especially proactive communication (4). Results here clearly supported that patient-centered care differentiated itself from the traditional perspective of doctor-patient communication, albeit the high correlation (*r* = 0.753) between the two concepts.

When comparing the association of PCC and patient satisfaction across different medical departments, the prediction of PCC scores onto overall satisfaction was stronger in Pediatrics than Surgery department. This is reasonable as pediatrics serve a particularly special and vulnerable population, namely children who may be uncapable to clearly communicate symptoms and needs, even for those accompanied by their caregivers (39). A PCC approach, e.g., characterized by empathy and patient's agenda, could possibly help ease the unfavorable anxiety, improves communication efficiency, and avoid conflicts with patients and their family (40).

No doubt that PCC makes considerable contributions to the improvement of overall satisfaction, yet this does not deny the critical practitioner-controlling role in life-threatening medical situations (41). In other words, there is no one-size-fits-all healthcare service approach, and no one research method or survey tool is inherently better than another. We suggest that the adoption of different healthcare approaches should be dynamic and mutual. Healthcare professionals might apply population (e.g., age group) by situation (e.g., urgency and severity) analysis and decide accordingly which approach to adopt, while healthcare institutions should integrate different care approaches in onboard training for their employees and diversify relevant performance evaluation systems.

### Limitation and strength

The generalizability of our findings was somewhat limited as we only recruited patients from one comprehensive hospital, despite that our selection was based on its representative city and population size in southern China. Granted which, this study introduced the concept of PCC in China to evaluate patient satisfaction which furthered the scope of different healthcare perspectives. Additionally, this study provided the Chinese version of the PPIQ scale as a feasible assessment tool for further studies despite the lack of discriminant validity of its factors. Finally, this study shed light on the unique contribution of patient-centered care in addition to other medical perspectives and discussed how healthcare professionals and institutions might make the best use of it in real settings.

## Conclusion

The Chinese version of the PPIQ scale (PPIQ-C) exhibited excellent internal consistency and acceptable content validity. Patient-centered care (PCC), reflected by the PPIQ-C scores, predicted overall patient satisfaction above and beyond other medical service perspectives. Adopting PCC approach in appropriate situations will probably advance the development of performance evaluation systems in China, thus improving the overall healthcare and patient satisfaction.

## Data availability statement

The original contributions presented in the study are included in the article/Supplementary material, further inquiries can be directed to the corresponding authors.

## Ethics statement

The studies involving human participants were reviewed and approved by the committee on research involving human subjects of School of Public Health, Shanghai Jiao Tong University School of Medicine. The patients/participants provided their written informed consent to participate in this study.

## Author contributions

ZZ, XuL, YF, KQ, and XiL: study design and implementation. SL: methodology. TH, SL, and XuL: data curation. TH and SL: analysis, validation, and writing-original draft preparation. CY, JL, TJ, and MB: discussion. HL and ZZ: supervision. All authors have read and agreed to the published version of the manuscript.

## Funding

This study was supported by a grant from Nanjing Jialiang Information Technology Co., LTD to ZZ.

## Conflict of interest

The authors declare that the research was conducted in the absence of any commercial or financial relationships that could be construed as a potential conflict of interest

## Publisher's note

All claims expressed in this article are solely those of the authors and do not necessarily represent those of their affiliated organizations, or those of the publisher, the editors and the reviewers. Any product that may be evaluated in this article, or claim that may be made by its manufacturer, is not guaranteed or endorsed by the publisher.
